# Basilar impression presenting as intermittent mechanical neck pain: a rare case report

**DOI:** 10.1186/s12891-015-0847-0

**Published:** 2016-01-11

**Authors:** Firas Mourad, Giuseppe Giovannico, Filippo Maselli, Francesca Bonetti, César Fernández de las Peñas, James Dunning

**Affiliations:** Alumno de Doctorado, Escuela Internacional de Doctorado, Universidad Rey Juan Carlos, Alcorcon, Madrid Spain; Fisioterapia Terapia Manuale, Lecce, Italy; Physioup, Rome, Italy; DINOGMI, Genova University, Genova, Italy; SSR Puglia INAIL, Bari, Italy; Alabama Physical Therapy & Acupuncture, Montgomery, AL USA; Nova Southeastern University, Ft. Lauderdale, FL USA; Universidad Rey Juan Carlos, Madrid, Spain

**Keywords:** Neck pain, Basilar impression, Craniovertebral congenital anomalies, Red flags, Physiotherapy

## Abstract

**Background:**

Neck pain is one of the most common musculoskeletal disorders in clinical practice. However neck pain may mask more serious pathology. Although uncommon in most musculoskeletal physiotherapy practices, it is possible to encounter rare and extremely life-threatening conditions, such as craniovertebral congenital anomalies. Basilar invagination is an abnormality where the odontoid peg projects above the foramen magnum and is the commonest malformation of the craniocervical junction. Its prevalence in the general population is estimated to be 1 %. Furthermore, it is a well-recognised cause of neck pain insomuch as it can be easily overlooked and mistaken for a musculoskeletal disorder. Diagnosis is based on the patient’s symptoms in conjunction with magnetic resonance imaging (MRI). If life-threatening symptoms, or pressure on the spinal cord are present, the recommended treatment is typically surgical correction.

**Case presentation:**

This case report describes the history, relevant examination findings, and clinical reasoning used for a 37 year old male who had the chief complaint of neck pain and occipital headache. After the history and the physical examination, there were several key indicators in the patient’s presentation that appeared to warrant further investigation with diagnostic imaging: (1) the drop attack after a triggering event (i.e., heading a football), (2) several episodes of facial numbness immediately and shortly after the trauma, (3) the poorly defined muscle upper extremity muscle weakness, and (4) the modification of symptoms during the modified Sharp-Purser test.

Therefore, the decision was made to contact the referring neurosurgeon to discuss the patient’s history and his physical examination. The physician requested immediate cervical spine MRI, which revealed a “basilar impression”.

**Conclusion:**

This case report highlights the need for more research into a number of issues surrounding the prevalence, diagnosis, and the central role of primary care clinicians such as physiotherapists. Furthermore it underlines the importance of including Basilar invagination in the differential diagnosis. Physiotherapists working within a direct access environment must take a comprehensive history and be capable of screening for non-musculoskeletal medical conditions (on a systems, not diagnosis level) in order to avoid providing potentially harmful musculoskeletal treatments (e.g., cervical mobilization or manipulation, stretching, exercise) to patients with sinister medical pathologies, not benign musculoskeletal disorders.

## Background

Neck pain is one of the most common patient complaints in clinical practice. It is estimated that up to 70 % of the population suffer from neck pain sometimes in their lives [1, 2]. Although the natural history of neck pain tends to be favourable in the acute phase, as primary care clinicians, physical therapists must pay attention on the presence of any red flags [3, 4].

A variety of causes of neck pain have been described and include osteoarthritis, discogenic disorders, trauma, tumors, infection, myofascial pain syndrome, torticollis and whiplash [5]. However clearly defined diagnostic criteria have not been established for many of these entities [6]. Similar to low back pain, patho-anatomical causes are not identifiable in the majority of patients who suffer from neck pain [7]. Therefore, once serious medical pathology has been ruled out, patients with neck pain are often classified by physiotherapists as having either neck pain with mobility deficits, neck pain with headaches, neck pain with movement coordination impairments, or neck pain with radiating pain [6].

Nevertheless, in a much smaller percentage of patients, the cause of neck pain may be due to more serious conditions such as fracture, malignant tumour, vascular disorders, or systemic disease [6].

Basilar invagination (BI) is a rare malformation of the craniocervical junction and is a well recognised cause of neck pain (NP), cough headache, lower cranial nerve palsies, corticospinal signs, hydrocephalus, cerebellar dysfunction, syringomyelia and syringobulbia [8–11]. Primary BI is a congenital abnormality associated with vertebral defects (Klippel-Fiel Syndrome, odontoid abnormalities, atlanto-occipital fusion, and atlas hypoplasia). The true prevalence of this condition is unknown but it is estimated to be less then 1 % in the general population [12]. Secondary BI, is less common and is caused by a developmental condition attributed to softening of the osseous structures at the base of the skull [13].

Diagnosis is made when the tip of the odontoid process crosses above the Chamberlain’ s line (a line traced from the posterior margin of the hard palate to the dorsal margin of the foramen magnum) [14].

Congenital, basilar invagination may remain asymptomatic and unrecognized until adulthood [15]. However, symptoms commonly present when the brainstem is compressed by the clivus or by the odontoid peg. When symptoms progress and threaten disability, treatment is by surgical decompression or by transoral odontoidectomy with reduction of the basilar impression and craniovertebral junction by bony realignment and atlanto-axial fixation [16, 17]. In fact, when preoperative dynamic neuroradiological examinations demonstrate that the cranio-vertebral junction compression is “reducible” during neck extension, neural decompression is suggested by the transoral or transnasal route in order to reduce the disclocation [18, 19]. In cases in which an accurate preoperative and intraoperative dynamic maneuvers and traction demonstrate the atlantoaxial dislocation “irreducibility”, the decompression may be obtained by stabilizing the cranio-vertebral junction with a posterior instrumentation [20].

Physiotherapists routinely assess patients whose primary complaint is NP alone. This case report highlights the importance of including Craniovertebral congenital anomalies (CVCA) in the differential diagnosis. Recognition of the reg flags associated with CVCA in the hypothesis generation phase requires knowledge of pathophysiology, clinical presentation and diagnostic testing. Failure to correctly screen for (i.e., the physiotherapist) or diagnose (i.e., the medical physician) CVCA could place the patient at risk of severe disability and possible permanent spinal cord damage [16]. The purpose of this case report is to increase clinicians’ awareness of the signs and symptoms of CVCA that can manifest as non-specific mechanical NP, and describe the assessment, screening and diagnostic process.

## Case presentation

### Case presentation

A 37-year-old man presented to the clinic with the chief complaint of intermittent neck pain, with no associated history of recent or past major trauma.

He also complained of spontaneous posterior head and occipital pain and stiffness during certain neck movements.

His current resting baseline pain level was 3/10 on a Numeric Rating Pain Scale (NPRS) (0, no pain;10, maximal pain) and his worst pain in past 24 h was reported to be 6/10 [21].

He was concerned because of one recent drop attack episodes after a header during a soccer match with subsequent facial numbness reported during prolonged postures after that incident.

The patient had not previously visited a medical physician or physiotherapist for these symptoms; however, he did admit controlling symptoms with the use of nonsteroidal anti-inflammatory drugs (NSAIDs) and pain killers.

From this episode he described a new spontaneous headache episode and limited neck range of motion (ROM) into right rotation. Additionally, he complained of dizziness during neck extension movements. He denied any night pain and reported no upper limb symptoms. However he described two previous episodes in which he had difficulties in lifting or pulling heavy weights. He wasn’t able to exactly define when the NP symptoms originally started. No other neurological symptoms were reported.

Review of the past medical history, including a review of symptoms, was performed. The patient did not have any significant past or current medical problems but was noted to not be concerned about the symptoms; however, he was seeking treatment for his occipital headaches that reportedly were getting more frequent and more intense.

### Examination

The physical examination started with visual analysis of posture, followed by active ROM testing of the cervical spine. Assessment revealed decreased active cervical ROM in all six planes, especially during left side bending and right rotation, with a dizzy feeling and facial numbness during sustained neck extension. During active cervical extension and right rotation, the patient also complained of occipital pain. No additional provocative or over-pressure testing were performed.

Cranial nerve testing (CNs II-V, VII-VIII, X-XII) were recorded as normal. There was no nystagmus, facial asymmetry, deviation of the tongue, or slurring of words.

Due to the absence of CN’ findings and the presence of dizziness and facial numbness during sustained extension, it was reasonable to pursue additional physical examination procedures in order to clear the cervical spine.

A more comprehensive set of neurological tests was performed. Upper extremity reflexes were found to be bilaterally present and symmetrical. No deficits were noted upon light touch sensory testing in the dermatomes of the upper extremities. However motor strength of the upper extremity muscles, assessed with manual muscle testing, revealed a 4/5 grade bilaterally on the C5 (shoulder abduction) innervated muscle group [22]. Ankle clonus was not present and Hoffman’s reflex [23] was negative. Rhomberg’s test did not reveal any loss of balance [24, 25].

Due to the presence of dizziness, facial numbness, focal muscle weakness in one myotome, and a recent drop attack episode, it was thought that his signs were more suggestive of a serious medical pathology, rather than a benign musculoskeletal condition.

However the examination continued with the assessment of the craniocervical structures. A modified Sharp-Purser test [11] was performed with the patient seated and the head brought into extension. The palm of one hand is placed on the patient’s forehead while the spinous process of the axis is held by the opposite hand. No discernable movement or reduction was noted but the positioning itself appeared to reduce the severity of the dizziness and facial numbness.

Several components of this patient’s history and physical examination were consistent with a condition for which physical therapy intervention would not have been indicated until more definitive cervical spine diagnostic imaging had been completed. More specifically, the physical therapist was primarily concerned about the possibility of a serious pathology that would preclude the use of manual therapy and/or exercise to craniovertebral region.

A decision to refer the patient to a neurosurgeon was made at this time. Magnetic resonance imaging (MRI) was deemed necessary prior to initiating any additional examination or treatment procedures.

### Diagnostic imaging

**Fig. 1:**
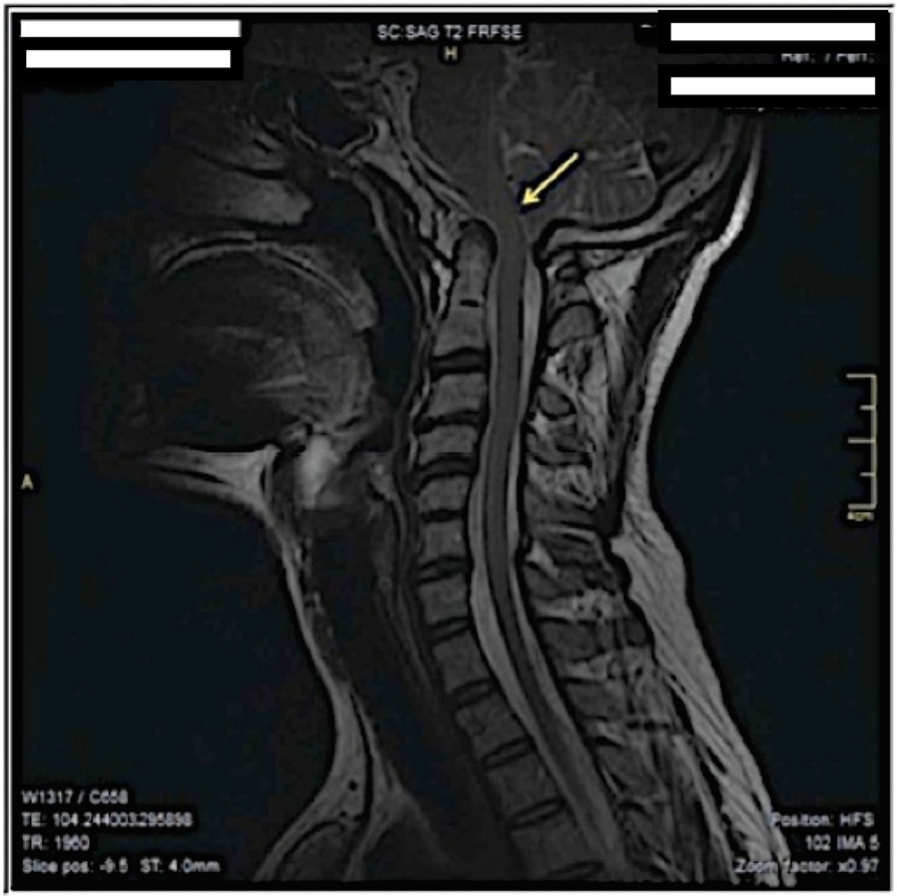
Sagittal T2-weighted MRI. The MRI of the Cranial-cervical structures showing the dorsal increase of the odontoid dens

**Fig. 2:**
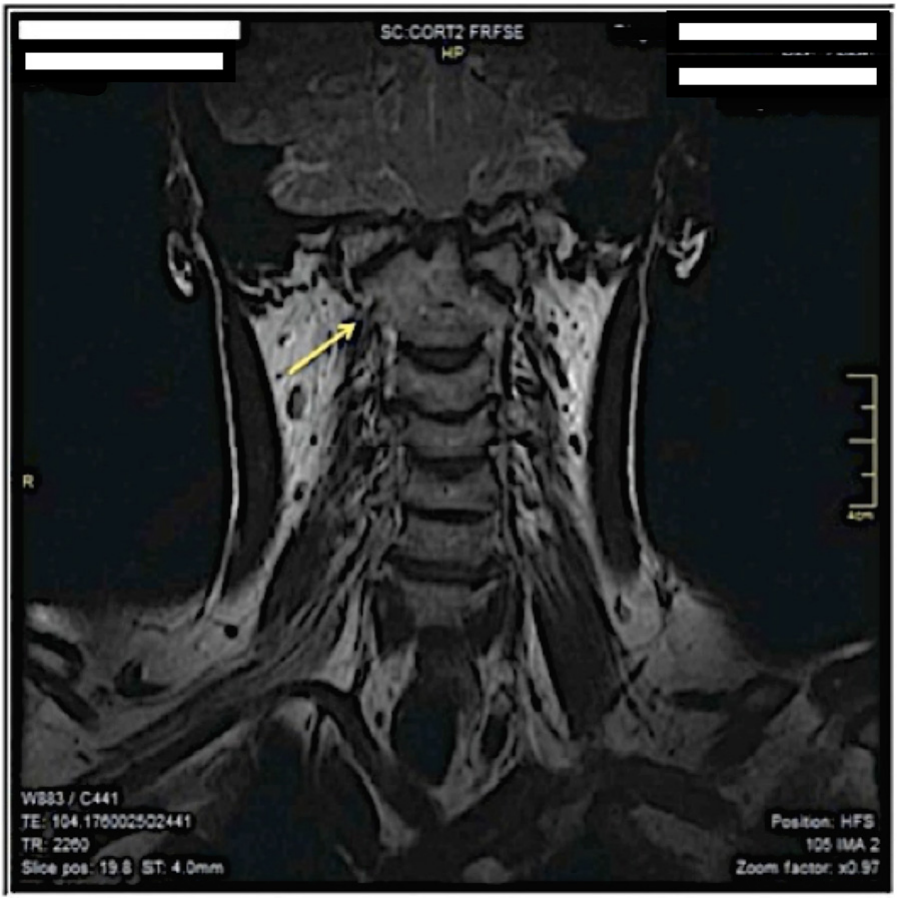
Coronal T2-weighted MRI. The MRI showing the cranial-cervical malformation with a subtotal fusion of the atlas with the occipital bone

A cranial-cervical spine MRI revealed a “basilar impression” in the sagittal T2-weighted image and demonstrated a slight dorsal increase of the odontoid dens with a cranio-dorsal sagittal shift of approximately 6 mm with associated footprint of the anterior surface of the bulbospinal tract (yellow arrow in Figs. 1 and 2). However, no areas of altered signal intensity of the medulla were identified. A reduction in amplitude of the foramen magnum was also noted, in which the cerebellar tonsils were wedged. Posterior somatic osteophytes, especially at C3-C4 were evident. A moderate disc protrusion at C3-C4 and at C4-C5 and C5-C6, without spinal cord compression, were also visualized [10] (Fig. 1). A coronal T2-weighted image demonstrated a cranial-cervical malformation with a subtotal fusion of the atlas with the occipital bone and a hypertrophic right articular process of the dens of the axis (yellow arrow in Figs. 1 and 2) resulting in an asymmetrical position of the facet joints (Fig. 2). A partial somatic fusion at C5-C6 was also visualized [26].

The neurosurgeon decided to not evaluate the dynamic stability [27] at the time.

## Conclusion

The aim of this case report was to discuss the relevant concepts relating to the pathophysiology, screening and differential diagnosis of a rare pathologic upper cervical disorder, and to further describe the relevant findings from the history and physical examination from the physiotherapist’s perspective in a patient with an unusual symptom presentation.

The term “basilar impression” often called “basilar invagination” is generally used to broadly label different types of deformities involving the posterior cranial fossa and the upper cervical vertebra. BI is an uncommon syndrome that occurs when the upper part of the odontoid dens migrates upward towards the foramen magnum [12]. The main etiologies of BI are clivus (basiocciput) hypoplasia, occipital condyle hypoplasia, atlas hypoplasia, atlanto-occipital assimilation and congenital atlanto-axial instability [14, 15, 28, 29]. Several neural axis abnormalities are commonly described in association with BI, especially Chiari type I malformation [15]. Chiari malformation and BI have much in common but are described as different clinical entities.

According to Goel et al. [30] the most common signs and symptoms included weakness (100 %), neck pain (59 %), posterior column dysfunction (39 %), bowel and bladder disturbance (28 %), and paresthesia (25 %). Localized findings included restricted neck movements (59 %), low hairline (48 %), webbed neck (47 %), and short neck (41 %). Notably, as in our case report, trauma is the principal precipitating factor in initiating symptoms in 48 % of patients with basilar BI.

This case report describes the clinical picture and clinical decision making that led a physiotherapist to suspect a non-musculoskeletal cause in a patient with NP, occipital headache, and dizziness.

This case also supports the concept that physiotherapists are trained and capable of screening for pathologic medical conditions in direct access settings; that is, physiotherapists are able to identify when a patient’s signs and symptoms are outside of the physiotherapy scope and in need of appropriate medical referral for further examination or investigation [31, 32]. Any suspicion led to the physical therapist contacting the referring physician to suggest the need for additional diagnostic imaging studies that lead to the best therapeutic treatment and prognosis for those patients at risk of serious pathologies.

Thus, a better understanding of the pathophysiology of this complex anatomical junction leads to a better understanding of the surgical management also [33]. Physiotherapists should to be aware that a regrowth of the odontoid process after odontoidectomy surgery is possible and a continuous postoperative neuroradiological examinations must be scheduled [34] during a post surgery management when indicated.

### Ethics approval

This study was approved by the Human Subjects Commitee of the Hospital Universitario Funacion Alcorcon and Universidad Rey Jaun Carlos, Madrid, SPAIN.

Human Subjects Committee Approval Letter 2014-12-55.

### Consent

Written informed consent was obtained from the patient for publication of this Case report and any accompanying images. A copy of the written consent is available for review by the Editor of this journal.

## Abbreviation

BI: Basilar impression
CNs: Cranial Nerves
CVCA: Craniovertebral congenital anomalies
MRI: Magnetic resonance imaging
NP: Neck pain
NPRS: Numeric pain rating scale
NSAIDs: nonsteroidal anti-inflammatory drugs
